# The 40-Something randomized controlled trial to prevent weight gain in mid-age women

**DOI:** 10.1186/1471-2458-13-1007

**Published:** 2013-10-25

**Authors:** Lauren T Williams, Jenna L Hollis, Clare E Collins, Philip J Morgan

**Affiliations:** 1 Nutrition and Dietetics, School of Health Sciences, Faculty of Health and Medicine, The University of Newcastle, 2308, Callaghan, NSW, Australia; 2 Nutrition and Dietetics, Faculty of Health, The University of Canberra, 2601, Bruce, ACT, Australia; 3 The Priority Research Centre in Physical Activity and Nutrition, The University of Newcastle, 2308, Callaghan, NSW, Australia; 4 School of Education, Faculty of Education and Arts, The University of Newcastle, 2308, Callaghan, NSW, Australia

**Keywords:** Obesity prevention, Intervention, Menopause, Motivational interviewing

## Abstract

**Background:**

Obesity prevention is a major public health priority. Despite the health risks associated with weight gain, there has been a distinct lack of research into effective interventions to prevent, rather than treat, obesity particularly at high risk life stages such as menopause in women. This paper describes the rationale for and design of a 2-year randomized controlled trial (RCT) (the 40-Something Study) aimed at testing the feasibility and efficacy of a relatively low intensity intervention designed to achieve weight control in non-obese women about to enter the menopause transition.

**Methods and design:**

The study is a parallel-group RCT consisting of 12 months of intervention (Phase 1) and 12 months of monitoring (Phase 2). Non-obese pre-menopausal healthy females 44–50 years of age were screened, stratified according to Body Mass Index (BMI) category (18.5-24.9 and 25–29.9 kg/m^2^) and randomly assigned to one of two groups: motivational interviewing (MI) intervention (n = 28), or a self-directed intervention (SDI) (control) (n = 26). The MI intervention consisted of five consultations with health professionals (four with a Dietitian and one with an Exercise Physiologist) who applied components of MI counselling to consultations with the women over a 12 month period. The SDI was developed as a control and these participants received print materials only. Outcome measures were collected at baseline, three, 12, 18 and 24 months and included weight (primary outcome), waist circumference, body composition, blood pressure, plasma markers of metabolic syndrome risk, dietary intake, physical activity and quality of life. Analysis of covariance will be used to investigate outcomes according to intervention type and duration (comparing baseline, 12 and 24 months).

**Discussion:**

The 40-Something study is the first RCT aimed at preventing menopausal weight gain in Australian women. Importantly, this paper describes the methods used to evaluate whether a relatively low intensity, health professional led intervention will achieve better weight control in pre-menopausal women than a self-directed intervention. The results will add to the scant body of literature on obesity prevention methods at an under-researched high-risk life stage, and inform the development of population-based interventions.

**Trial registration:**

ACTRN12611000064909

## Background

Obesity prevention in adults has the potential to significantly decrease the burden of disease in the population and costs to the health system [1,2]. Prevention of weight gain is particularly important at life stages where fat is deposited abdominally, since this confers additional health risks by contributing to metabolic syndrome [3,4]. Longitudinal studies have shown that middle age (45–54 y) is a high risk stage for weight gain in Western Women [5-8]. Prior to the onset of menopause, women have a low prevalence of cardiovascular disease and associated risk factors [9]. The physiological change initiated by menopause alters this level of risk, primarily through the preferential deposition of fat abdominally due to oestrogen deficiency [5,10-12]. National data show the prevalence of abdominal overweight and obesity in Australian women jumps from 66.8% in the 45–54 year age group to 77.9% in the 55–64 year age group [13], with the latter group having a dramatically higher increase in cardiovascular disease [14]. An estimated 48% of coronary events in women are attributable to metabolic syndrome [15]. Weight gain after menopause also increases the risk of breast cancer [16].

While there is a substantial body of evidence showing that weight loss in the obese reduces cardiovascular risk, the body of evidence on weight gain prevention interventions is scant [17], with only one RCT investigating whether weight gain prevention in pre-menopausal women can mitigate the increase in cardiovascular risk usually associated with menopause [18,19]. The Women’s Healthy Lifestyle Project conducted in the United States was designed to prevent weight gain and increased cardiovascular risk associated with menopause [18,19]. Pre-menopausal women (44–50 y, N = 535) randomized into a lifestyle intervention group (15-session diet and exercise education program followed by a maintenance program) lost on average 0.1 (5.2) kg after 54 months compared with a gain of 2.4 (4.9) kg in the assessment-only group [19]. While the trial showed that it was possible to prevent menopausal weight gain and to limit the typical post-menopausal rise in low density lipoprotein (LDL) cholesterol, the researchers acknowledged that the 15-session intervention held over 20 weeks was labour intensive and required a substantial time commitment from participants [20]. There is a need for the development of less intensive interventions that could feasibly be implemented on a population basis.

While women are known to attempt weight control (defined as weight maintenance for those of healthy weight and weight loss for the overweight) [21], self-directed efforts have not prevented population weight gain. Data from over 12,000 mid-age women in the population-wide Australian Longitudinal Study on Women’s Health showed 70% of participants reported behaviours aimed at losing weight or preventing weight gain, but they were mostly unsuccessful [21]. In the first two years of the study, the women without surgical menopause gained a mean (SD) of 1.04 (4.7) kg [22], and the cohort continued to gain weight at 0.5 kg/year over the next three years [23]. In a study of the weight control practices used by the women [21] the only group who managed to successfully prevent weight gain included a commercial program in addition to the typical public health strategies of 'cutting down on the size of meals and snacks plus cutting down on fats and sugars plus exercise’. This demonstrates the need for population based interventions that provide the face-to-face support typical of commercial programs, in addition to the more traditional awareness raising and education strategies.

In Australia, a model utilising face-to-face health practitioner support with potential to be implemented nationally already exists within the healthcare system but has not been used with this target group. The Chronic Disease Management scheme of Medicare Australia funds five consultations with allied health professionals in a 12 month period for those referred by a primary care physician with chronic conditions and complex medical problems [24]. This program could potentially be extended to women on the cusp of menopause, given it provides face-to-face contact but is still relatively low intensity. The addition of MI as a client-centred counselling technique to the health professional consultations provides the opportunity to strengthen commitment to behaviour change by reducing resistance to change and enhancing intrinsic motivation [25] with evidence of improved quality of nutrition and physical activity counselling [26]. The efficacy of this model in preventing weight gain in pre-menopausal women needs to be tested. In fact, no interventions to prevent menopausal weight gain have been tested in Australian women to date.

### Study aim

This paper describes the research protocol for an RCT (the 40-Something Study) aimed at testing the feasibility and effectiveness of applying a health professional led, weight gain prevention intervention in non-obese women about to enter the menopause transition (aged 44–50 years). The feasibility of recruiting to the study, ability to retain participants and the suitability of the written materials were tested along with the preliminary effectiveness of the intervention. There are two hypotheses:

1. Women in the MI group will lose at least 2.5 kg by the end of the intervention (12 months post-baseline) and maintain that loss for a further 12 months (to 24 months post-baseline).

2. Women in the SDI (control) will gain 0.5 kg by 12 months and 1.0 kg by 24 months.

## Methods/design

### Study design

**Figure 1:**
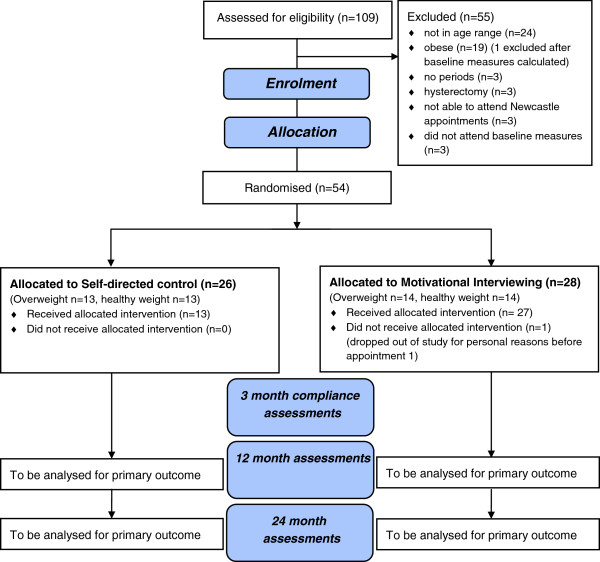
**CONSORT Flow chart describing the progress of participants through the trial**[28]**.**

The study design is a parallel-group RCT as shown in Figure 1. Weight change outcomes for the intervention provided by health professionals utilising MI will be compared with equivalent advice provided in written format (SDI). At the conclusion of the 12-month intervention (Phase 1), goals will be reassessed and participants entered into a 12-month monitoring phase (Phase 2) to determine whether intervention effects can be sustained. The University of Newcastle’s Human Ethics Research Committee approved this study (approval number H-2010-0030). Written informed consent was obtained from all participants prior to enrolment. The RCT was designed to adhere to the Consolidated Standards of Reporting Trials (CONSORT) guidelines [27,28] and the trial protocol was registered with ACTRN12611000064909. Following eligibility screening (using criteria shown in Table 1), weight and height were measured by researchers to calculate BMI, with participants categorised as Healthy weight: 18.5 to 24.9 kg/m^2^ or Overweight: 25–29.9 kg/m^2^. Equal numbers of women in each BMI category were then randomised to the MI or the SDI.

**Table 1:** Eligibility criteria for the 40-something study

***Inclusion criteria***	***Exclusion criteria***
Female	Hysterectomy and or oophorectomy
BMI between 18.5 and 30 kg/m^2^	Pregnant
Menstrual period within preceding 3 months	Taking hormone replacement therapy
Born in the years 1960 to 1966	Reported diagnosis of cardiovascular disease, diabetes, cancer
Able to attend appointments at the University	
Able to communicate in English	

### Participants and recruitment

Non-obese (BMI between 18.5 and 30 kg/m^2^) pre-menopausal women aged 44–50 years were recruited from the region surrounding Newcastle, NSW, Australia between May and July of 2010. Recruitment channels included radio and television interviews, newspaper articles and flyers placed in medical clinics, and University notice boards. Women expressing interest in the study contacted researchers and were screened for eligibility over the telephone by the first two authors according to a documented protocol based on the inclusion and exclusion criteria shown in Table 1. Weight and height were self-reported by the women and BMI calculated. Due to the limitation of self-reported height and weight, the BMI criterion was re-checked using laboratory measurements at the first assessment visit, and anyone who had misreported or underestimated their weight to the extent that their BMI was in the obese range was subsequently excluded. One woman was found to be obese (BMI over 30 kg/m^2^) at this stage. Participants not meeting all of the eligibility criteria during telephone and laboratory screening were excluded for the reasons shown in Figure 1.

### Randomization, blinding and quality assurance

Randomization was achieved using a computer-based random number sequence. Once written consent was obtained, participants were enrolled in the study. Baseline assessments were then completed and BMI status was checked and the women were categorised as healthy weight or overweight. At the end of the first assessment the enrolled women were given a participant identification number by a researcher (LTW). The numbers used were from a random allocation sequence generated by a statistician using a computer-based random number-producing algorithm to ensure an equal chance of allocation to each intervention group (the Plan procedure (Proc Plan) in the SAS statistical software package) in block lengths of six. The sequence was divided into two separate lists — one for each BMI category. LTW checked the BMI category of each participant at the conclusion of the first assessment, then allocated the participant to the next vacant number sequence on either the healthy weight list or the overweight list as appropriate. This ensured approximately equal numbers of healthy weight and overweight women were assigned to each intervention group. LTW remained blinded to group assignment. A separate version of each list, with intervention group appearing next to each identification number was only given to one researcher (JLH) who was unable to remain blinded after baseline measures since she delivered the face-to-face Dietitian consultations. She phoned the women within a day of their baseline measures to inform them of their group assignment.

All other researchers and research assistants remained blinded to group assignment throughout the study. When presenting for subsequent assessments, participants were reminded not to reveal their assignment group to the researchers and research assistants. To minimise the effects of the potential bias of JLH being unblinded, this researcher only assisted with automated measures at 3 months. However, at 12 and 24 months JLH was required to collect waist circumference measures for measurement consistency with the baseline collection of that measure. To confirm success of researcher blinding an audit tool was constructed and administered at the end of each measurement session. All assessors recorded which group they believed the participants were assigned to (MI or SDI), and indicated the reason for their answer from a list of tick box items, which included a) just a guess, b) participant revealed it to me directly, c) participant revealed it to me indirectly, d) based on the participant results, or e) other reason, with space provided to record the 'other’ answer. The project was monitored by a Research Management Committee who met monthly to oversee the implementation of the project.

### Goals of the intervention

Both the MI and SDI women were given intervention goals according to their baseline BMI as shown in Additional file 1. Women found to be in the healthy weight range (18.5 – 24.9 kg/m^2^) at baseline were advised to maintain that weight within 1 kg for the duration of the study. Written advice was provided in booklet format so the women could meet their Recommended Dietary Intakes (RDI) for micronutrients and macronutrients [29] and an intake of 8300 kJ/day, which is the estimated requirement for women 51–70 years with a mean height of 1.6 m and an activity factor of 1.6 [29]. The booklet also outlined how to meet physical activity recommendations of at least 150 mins/week of moderate intensity physical activity [30], 10000 steps per day [31] and restriction of sitting time to 3 hr/day or less [23,32].

Women with a baseline BMI in the overweight range (25–29.9 kg/m^2^) were advised to lose up to 7% of baseline weight (an amount shown in previous interventions to have significant clinical outcomes for the overweight) [33]. Written advice was provided in booklet format to meet the same RDIs as the healthy weight women, within a maximum energy intake of 6300 kJ/day, which is 2000 kJ less than the estimated requirement for weight maintenance of 8300 kJ [29] in order to achieve a weight loss of approximately 0.5 kg per week. Physical activity advice was increased to 250 mins/week of moderate intensity to help achieve weight loss [34], with the same daily step count and sitting time as the healthy weight group.

A protocol responding to changes in BMI category was developed. It stated that, if in achieving their weight loss goal the overweight women achieved a BMI in the healthy weight range, they were advised to maintain that new weight within 1 kg for the remainder of the study and would be given the written booklet containing advice prepared for healthy weight women. Conversely, any healthy weight women found to have gained sufficient weight to be reclassified as overweight would be given the written advice and behavioural goals for the overweight women, and advised to lose weight to re-attain the healthy weight range. The process for change in goals is shown in the Figure in Additional file 1.

### Sample size

The sample size calculation was based on the main outcome measure of body weight. Observational studies of women in this age group in the Australian population have demonstrated a mean (SD) weight gain of 1.04 (4.7) kg over a two-year period [22]. Given both intervention groups were comprised of equal numbers of healthy weight and overweight women and presuming the overweight women would meet the intervention goal of losing 5 kg^a^ in the first 12 months and the healthy weight women would show zero weight change, the net weight change should be a 2.5 kg weight loss in the MI group by the end of phase 1. Based on the Australian data of net weight gain in the general population of mid-age women managing their weight by self-directed means [21], we would expect a mean (SD) net weight gain of 0.5 (4.7) kg per year in our SDI (control) group. This would give an expected difference between the two groups of 3.0 kg after 12 months (end phase 1) and 3.5 kg after 24 months (end phase 2). Assuming equal sample sizes, to detect a difference in weight between groups of 3.5 kg with 80% power and a significance level of 0.05, a sample size of 22 women was required for each group. Allowing for an attrition rate of 20% by the end of 24 months, a total sample size of 55 women is required.

### Theoretical framework and intervention materials

Weight gain prevention requires permanent changes to dietary intake and physical activity behaviours. Cognitive and behavioural psychology provides models to inform strategies aimed at achieving such change. The interventions were framed according to the principles of Social Cognitive Theory (SCT) [35]. SCT aims to provide an understanding of why and how people change individual health behaviours within the social and physical environments that affect these behaviours [36]. Intervention materials (weight control booklets, documented goals and strategies, weight and behaviour monitoring tools and the consultation protocols) were purpose-developed and incorporated elements of SCT. The weight control booklets and weight and behaviour monitoring tools given to participants in both conditions empowered participants to self-monitor. Two versions of the weight control booklets were developed: one for women in the healthy weight range at baseline providing weight maintenance advice, and one for women in the overweight range at baseline providing weight loss advice.

Women in both groups received tailored, written weight control goals and diet and physical activity behaviour change goals and strategies that were developed individually for each participant by health professionals according to the results of their assessment. The documentation reiterated the weight control message, and then guided the participant on how to achieve change. For SDI participants not meeting recommendations, the behaviour change goal and strategy was documented on their goal sheet and mailed to them. For example, “Aim to eat 2 serves of fruit per day. Some suggestions of how you could achieve this may include: incorporate 1 serve of fruit into your afternoon snack, try adding dried or fresh fruit to your muesli and yoghurt for breakfast, try having 1 serve of fruit with yoghurt for dessert”. MI participants were empowered to negotiate their own goals and strategies with a health professional in a face to face consultation. For example, the participant was asked to identify if increasing fruit intake was a behaviour they would like to change. If the participant agreed, they were asked to identify a realistic fruit goal (e.g. eating 1 serve of fruit per day). Next they were asked how they may go about achieving this goal. It was their responsibility to identify strategies to change their behaviour. If exhaustive exploration of their own ideas yielded no feasible strategies, the health professional sought consent to provide advice and recommended a suite of potential strategies from which the participant could choose.

The weight gain prevention advice was based on 10 messages (seven dietary intake messages and three physical activity messages - see Table 2) developed using available evidence on the factors contributing to weight gain in mid-age women [23,31,37-39] and national recommendations for this age group [30,40] as shown in Table 2. Messages were phrased using positive language seeking to enhance self-efficacy, which is the confidence to perform a specific behaviour [35]. The phraseology emphasised that change is a possibility, for example *“****You can avoid ****gaining weight during this life stage by…”.* The messages were pre-tested in an earlier study via telephone interview with a national sample of 40 women to ensure that they were clear, relevant and consistent with acceptable communication.

**Table 2:** **Ten messages aimed at preventing weight gain**^**1 **^**in mid-age**

***Target behaviour***	***Message wording***
1. Improve diet quality, limit non-fibre sources [37,39,40]	Aim to eat two serves of fruit every day with no more than one serve in the form of juice.
2. Improve diet quality [37,40]	Aim to eat at least five serves of vegetables every day
3. Improve diet quality [37,40]	Aim to eat one to one-and-a-half serves of meat or meat alternatives every day
4. Improve diet quality [37,40]	Aim to eat two to three serves of dairy every day
5. Increase dietary fibre [39]	Aim to eat wholegrain varieties of breads and cereals.
6. Decrease energy intake [37,40]	Limit your intake of extra foods, which are high in fat and sugar, to two serves per day or less
7. Decrease energy intake [38]	Aim to cut down on the number of meals eaten outside the home each week
8. Meet national physical activity recommendations [30]	Aim to engage in moderate to vigorous physical activity for at least 30 minutes, five days per week or 150 minutes total per week.
9. Decrease sedentary activity [23]	Aim to sit for less than three hours per day
10. Increase physical activity [31]	Aim to take at least 10,000 steps per day

^1^The messages for the overweight women were aimed at weight loss and differed in the following way: 1.5 serves per day or less’ Message 8: read '250 minutes’ [34].

The MI intervention was based on the model of health professional consultations in chronic disease management within the Australian health system. To be consistent with the SCT constructs of self-efficacy and outcome expectations, MI was chosen as the counselling style for the consultations with health professionals. MI is a client centred style of counselling first described by Miller and Rollnick in 1983 [25]. It aims to facilitate behaviour change through a guiding method of communication and in so doing provides an environment to encourage a collaborative partnership between the client and health professional where the client feels comfortable to explore and resolve ambivalence towards changing behaviour [25]. Specifically, client confidence in performing a specific behaviour was enhanced by exploring and drawing on client strengths and past successes with both behaviour change and other difficult times in their life. Strategies used to overcome adversity were identified by the client. The protocol encouraged the health professional to convey hope and confidence that change was possible during all consultations. The MI intervention assisted participants in identifying outcome expectations within the consultation by using a decisional balance activity, where participants were encouraged to identify both the advantages and disadvantages of making a change to their behaviour, and through open-ended questions such as “How might your life be different if you were to achieve your goal weight?”. The MI counselling style aims to explore both sides of ambivalence and enable to client to reflect on how their personal values and beliefs were inconsistent with their current diet and physical activity behaviours. This counselling style thereby aimed to reduce the perceptions of negative outcomes to change.

Various MI activities and communication skills (rolling with resistance, expressing empathy, developing discrepancies between current and desired behaviour, supporting self-efficacy), were incorporated into the consultation protocols (as described in Table 3) with the aim of assessing motivation levels, encouraging change talk and enhancing intrinsic motivation to change [25]. Protocols for the health professional consultations were developed by researchers from Clinical Best Practice Weight Management Guidelines [41] and the Australian Physical Activity Guidelines [30].

**Table 3:** Motivational interviewing protocol demonstrating how the MI principles were upheld in the health professional consultations

**MI concept**	**An example of how MI was upheld during the consultation**
Autonomy	The participant was encouraged to set or revise their own dietary and physical activity strategies with the guidance of the health professional, including developing and revising six specific, measurable, achievable, realistic and timely (SMART) dietary and exercise goals, negotiating a change plan and setting the agenda.
Collaboration	The health professional avoided advice giving and only provided advice when requested by the participant or after consent to provide advice had been sought (E.g. “would you like me to give you a suggestion of what has worked for other people in a similar situation to you?”). The participant’s permission was also sought when discussing the importance of and options for monitoring weight, diet and physical activity, helping to facilitate a productive discussion.
Evocation	The health professional asked the participant to explain what they value in life and how these values may help to boost motivation “Think about all aspects of your life and tell me the characteristics that are most important to you?” (E.g. my family). “What is it about those aspects of your life that you value?” (E.g. I enjoy spending time with my grandchildren). “How might the values you mentioned help motivate you to make changes to diet and exercise behaviours?” (E.g. I need to improve my health to make sure I am here to see them grow-up). The participant was asked to report how important weight control is to them. “On a scale of 1 to 10, with 1 being the lowest and 10 being the highest, how important is weight control to you?”. “What made you choose a 7 and not a lower number?” (E.g. I chose 7 because I value my health and fitness levels and I know that weight control is important for these). “What would it take to bump you up a few notches to a number 9?” (E.g. If I had a health complication).
Roll with resistance by avoiding arguments and confrontation	Shifting the focus (“I can hear that your drinking is not something you would like to talk about now. Is there something else you would like to talk about with the time we have today?”).
Express empathy by accepting the clients perspective without judging or criticising	Through the use of reflections such as “that must have been very difficult for you”.
Develop discrepancy between current behaviour and desired goals and values	Participants were asked to identify their desired goals and values during the consultation and were then asked to describe their current behaviour. The participant was encouraged to identify the discrepancy between their current behaviour and their desired goals.
Supporting self-efficacy and fostering a belief that a change is possible may increase confidence levels and increase motivation	Participant confidence was enhanced by exploring and drawing on client strengths and past successes with weight control and other difficult times in their life and the strategies they used to overcome the adversity. Participants were also asked to report on their confidence to make a change using a 10-point Likert scale. Barriers to change and strategies to overcome these barriers were identified.

The proficiency of health professionals in delivering consultations consistent with MI practices will be evaluated through the Motivational Interviewing Treatment Integrity (MITI) 3.0 scale [42] by a qualified psychologist trained in MI who is independent of the study. The MITI is a behavioural coding system that assesses how well a practitioner adheres to the spirit of MI by coding a 20-minute random segment of the consultation on global scores on evocation, collaboration, autonomy, direction and empathy and behaviour counts on questions, reflections and MI adherent and non-adherent communication. Our recent systematic review [43] of MI interventions for diet and physical activity behaviour change, has shown the literature to be largely lacking in evaluating MI integrity and reporting proficiency scores, yet these measures are essential in ensuring health professional competence in delivering the MI intervention. All Dietitian consultations were pilot-tested on one of the researchers and revised prior to delivery to participants.

### Protocol

#### ***Phase 1: Intervention delivery 0 to 12 months***

In phase 1 the women completed baseline assessments, were assigned to groups to receive the relevant intervention, were reassessed at 3 months for compliance to protocols, and a final assessment was conducted at 12 months. The intervention components are shown in Additional file 2.

1) MI group – five x 60 minute Health Professional consultations.

Within a month of baseline measures being taken, women in this group received a 1 hour consultation with a Dietitian (consultation 1), followed by a one hour consultation with an Exercise Physiologist (consultation 2) around the same time. They received three subsequent one hour appointments with the same Dietitian three, six and nine months later (consultations 3, 4 and 5). Each consultation followed a protocol incorporating individualised counselling, tailored according to assessment results, and was delivered using the principles of MI. The health professionals (Accredited Practising Dietitian and Exercise Physiologists) were registered Medicare Australia providers trained to promote changes in behaviour in order to achieve weight maintenance or weight loss goals. They had also undertaken additional training in the application of MI. The same Accredited Practising Dietitian (JLH) conducted all Dietitian appointments, and the Exercise Physiology consultations were shared between two practitioners. While attending consultations 1 and 3 the women were given print copies of their assessment results (biochemistry, anthropometry, diet and physical activity analysis) from baseline and 3 month measures respectively and received intervention support materials (weight control pamphlet relevant to their baseline BMI, a weight tracking sheet, menstrual record sheet, and a food and physical activity diary for self-monitoring) at consultation 1. Weight control goals and behaviour change strategies (three for diet and three for physical activity) were negotiated in writing during consultations.

2) SDI group (control) – written advice only

Within a month of baseline measures being taken, women in the SDI group received individualised advice tailored according to their assessments in written form through the postal service. Mail out 1 consisted of a welcome letter, their baseline assessment results (biochemistry, anthropometry, diet and physical activity analysis), written weight control goals and behaviour change strategies developed based on assessment results, and the same intervention support materials given to the MI group (weight control pamphlet relevant to their baseline BMI, a weight tracking sheet, menstrual record sheet, and a food and physical activity diary for self-monitoring). Mail out 2 occurred within a month of the three month measures and consisted of their 3 month assessment results, revised weight control goals and three behaviour change strategies each for diet and physical activity tailored to their three-month assessment results, and further copies of self-monitoring materials.

#### ***Phase 2 – monitoring phase – 12–24 months***

Both groups were treated identically in the monitoring phase. While attending their 12-month assessment, all participants were given the results of those measures (with the exception of their blood results which were mailed to them). Their weight goals were reset on the basis of their 12 month results, documented and given to the women prior to their leaving the laboratory at the end of the 12 month assessment visit, which was also the beginning of Phase 2. Participants received no additional intervention appointments and no new materials in the monitoring phase, but were encouraged to continue self-monitoring their menstrual status, weight and diet and physical activity. They attended measurement assessments at 18 and 24 months, and were asked not to reveal their group allocation to researchers at these visits.

### Outcome measures

Outcome measures were assessed for all participants at baseline, between May-September 2010, and reassessed at 3, 12, 18 and 24 months (end of monitoring phase 2) as shown in Additional file 3. The primary endpoints are at 12 months to determine the effects of the intervention and at 24 months to assess whether the intervention effects were maintained. Weight was the primary outcome measure.

#### ***Measurement procedure***

Assessments were conducted in the early morning, in the anthropometry laboratory at the University of Newcastle (Australia). Procedures and protocols for the measurements were standardised, documented, and research assistants were trained in their application prior to collection. Participants fasted overnight, and upon entry to the laboratory removed their shoes and excess clothing. The assessment process lasted approximately 60 minutes for each participant, and they were provided with breakfast prior to leaving the laboratory.

#### ***Anthropometry***

Height was measured using a stadiometer (Surgical and Medical Products) correct to 0.1 cm using the stretch stature method. Weight and body composition (percentage fat, skeletal muscle mass) were measured at all time points using BIA Omron HBR-500 Body Composition Monitor with Scales correct to 0.1 kg. BMI was calculated using the standard equation (weight [kg]/height[m]^2^). Waist circumference was collected as a valid marker of central adiposity [44] using a non-extensible steel tape to measure at the level of the mid-point between the lower costal border and the iliac crest (KDS F10-02, KDS Corporation, Osaka, Japan). Two measures were collected and averaged. Visceral adipose tissue area was estimated using the InBody720 Body Composition Analyzer (Biospace) at 12, 18 and 24 months.

#### ***Biomarkers of metabolic syndrome***

Thirty millilitres of blood was collected from each participant by a trained phlebotomist and analysed by a laboratory accredited with the National Association of Testing Authorities Australia. Fasting measures of total cholesterol, low density lipoprotein cholesterol, high density lipoprotein cholesterol, triglycerides and glucose were assessed. Blood samples have been stored in a -80°C freezer for further analyses (sex hormones, insulin, leptin, and antioxidants to validate dietary intake) should funds become available.

#### ***Blood pressure and resting heart rate***

Systolic and diastolic blood pressure and resting heart rate were measured after participants rested for at least five minutes using a NISSEI/DS-105E digital electronic blood pressure monitor (Nihon Seimitsu Sokki Co. Ltd., Gunma, Japan) under standardized procedures. Three measures were taken, with a two minute rest between each. The result for the first measure was discarded, and measures 2 and 3 averaged.

#### ***Dietary intake***

Dietary intake was measured using a weighed food record method over four consecutive days, including one weekend day. While attending the laboratory for assessment sessions at baseline, 3 months and 12 months, participants were provided with a diet and physical activity record booklet and trained by an Accredited Practising Dietitian to record their usual dietary intake into the booklet, which included detailed written instructions. Participants were provided with electronic kitchen scales correct to 0.1 g (Soehnle Siena Electronic Kitchen Scale; Soehnle, Germany) and were instructed to record the weight, in grams, of their food and beverage portions and any leftovers. Participants were encouraged to keep detailed descriptions of foods, snacks and drinks including alcohol and water, and to note the brand names of packaged foods, cooking methods, and whether food was prepared outside the home. The 4-day dietary intake data was checked for completeness by a Dietitian and entered into Foodworks 2009 Professional Edition software version 6 (Xyris Software [Australia] Pty Ltd), a nutrient analysis package that uses national food composition data. Results were analysed according to Australian standards for energy intakes and Nutrient Reference Values [29] and dietary compliance to core foods groups according to the Australian Guide to Healthy Eating [40].

#### ***Physical activity***

Step counts were measured using Yamax SW200 pedometers (Yamax Corporation, Kumamoto City, Japan). While attending the laboratory for assessment sessions at baseline, 3 months and 12 months, participants were provided with pedometers which were reset to zero, attached to participants (at the waist on the right hand side) then tested. Participants were asked to wear pedometers for the same four consecutive days as they recorded their food record and to maintain their normal routine. Participants were asked to remove the pedometers when sleeping, in water (e.g. swimming, showering), or during contact sports, and to record their steps in their four day diary and reset pedometers to zero at the end of each recording day. Physical activity and inactivity was assessed by participants recording in their diary the number of hours spent in light, moderate and vigorous physical activity and walking, and sedentary behaviour (sitting time and sleeping hours), assessed against an adapted version of the sitting questionnaire [45,46]. Participants completed the International Physical Activity Questionnaire (IPAQ) short 'last 7 d’ version which has been found to be both reliable and valid in assessing physical activity in adults [47,48]. The physical activity results were compared to the intervention goals of 150/250 minutes per week (healthy weight/ overweight), 10,000 steps per day [31] and less than three hours of sitting time [23].

#### ***Dietary restraint***

Dietary restraint was measured using the Three Factor Eating Questionnaire (TFEQ) at baseline, 12 and 24 months [49] to assess whether restraint predicts weight control success.

#### ***Quality of life***

Quality of life, specifically perceived physical and mental health, was measured at baseline, 12 and 24 months through administration of the Short Form 36 (SF-36) questionnaire, [50] which is a widely accepted measure of health and wellbeing. Component summary scores for physical and mental health were able to be interpreted in an Australian context calculated using a formula standardised to Australian women in the target age group [51].

#### ***Menopause status and symptoms***

Participants recorded, in a purpose-developed calendar provided, the number of days in which menstrual bleeding, including spotting, occurred for the 24 month duration of the study. There was also space for the women to make comment about their periods or menopausal symptoms. Women with no menstrual bleeding for ≥3 and <12 months were categorised as peri-menopausal; women with no menstrual periods for ≥12 months were categorised as post-menopausal [52]. This data was collected to assess stage of menopause transition at 12 and 24 months.

A questionnaire [53] on frequency (often, sometimes, rarely, never) of menopause symptoms (hot flushes, night sweats, headaches/migraine, heavy bleeding/flooding, backaches/joint soreness, mood swings, depression, dry eyes, vaginal dryness) in the past three months was included in the participant surveys at baseline, 12 and 24 months.

### Process evaluation

Intervention implementation was evaluated by assessing the reach of the study, how successfully the intervention was implemented, participant satisfaction with implementation materials and intervention delivery, and by measuring participant compliance with the intervention goals. Reach was assessed by collecting demographic and health data on participants at baseline. Implementation records were kept by two researchers, who met at least weekly to check the implementation plan was being adhered to and was progressing according to the timeline. A participant satisfaction and study feasibility survey was purpose-developed for each intervention group by adapting and adding to a process evaluation for another weight control intervention [54]. Participants were asked to complete rating scales about each intervention component and to provide open ended comments on the strengths and weaknesses of the program, how they felt about their random group assignment, how much they would be willing to pay for the offered intervention, and any suggestions for study improvement. The survey was completed by participants while attending the laboratory for 12 month measures. A modified version of the process evaluation survey was developed for the monitoring stage, and was completed by the women attending 24 month measures.

Compliance with weight, physical activity and dietary intake goals was assessed at three months using the data from the four day weighed food record, pedometer readings and the physical activity diary. A compliance score was developed for each of the 10 messages included in the weight control pamphlets. If a participant was fully compliant with a message they were given a score of 10, and if totally non-compliant they were given a score of one. For example, if a participant had eaten at least two serves of fruit each day, they were given a score of 10. The score would be proportionately lower for fewer than two serves of fruit daily with no fruit consumption resulting in a score of one. Each individual compliance score was totalled, giving a maximum possible score of 100 (indicating full compliance) and a minimum possible score of 10.

### Data cleaning and checking

Data was recorded originally in hardcopy then entered into a purpose built ACCESS database. All data entry was checked for accuracy by two researchers and any errors recorded and corrected. A Dietitian researcher checked the accuracy of FoodWorks data entry against the computed records prior to their entry into the ACCESS database.

### Data analysis

Outcome measures collected at baseline will be compared to those at 12 months (after adjusting for baseline weight) to assess intervention effects and compared with measures at 24 months (end phase 2) to assess whether the intervention effects were maintained. Analyses will be conducted using the intention to treat principle, and performed using SPSS version 19 (IBM). All variables will be checked for plausibility. Continuous variables will be assessed for normality. Independent sample t-tests and chi-squared tests will be conducted on continuous and categorical variables (respectively) to check they do not vary by intervention group at baseline, and by completers versus attriters. Analysis of covariance will be used to examine whether the intervention groups differed significantly in weight at each of 12 and 24 months after adjusting for baseline weight. Behaviour changes in diet and physical activity will be assessed as secondary outcome measures using analysis of covariance to determine whether the MI and SDI groups differed significantly in dietary or physical activity behaviours at 12 months. Results will be stratified for baseline BMI category.

Logistic regression analysis will be performed to assess whether categorical variables, such as smoking status significantly influenced weight. The visceral adipose tissue variable will be assessed for between group differences at 12 and 24 months using independent sample t-tests, and analyses stratified by BMI category to determine whether the results differed for the healthy weight and overweight women. Sensitivity analyses will be conducted to determine the effects on the outcomes of different methods of treating loss to follow-up (last observation carried forward versus imputation). The relationship between weight outcomes and compliance with the diet and physical activity behaviours will be assessed using mediation analysis [55].

A per protocol analysis will be conducted on women who adhered to the requirements of their assigned group. Adherent women are defined as those who completed all required eating and physical activity diaries over the 12-month period and attended all assessment sessions, regardless of assigned group, and who attended all five health professional consultations for the MI group. Results of the protocol adherent group will be compared with non-adherers in each group, that is, those who did not meet the adherence recommendations.

## Discussion

The study described in this paper, the 40-Something study, is the first RCT aimed at preventing menopausal weight gain in Australian women and one of only a few internationally to do so. Importantly, this paper describes how an RCT of a relatively low intensity intervention, based on an existing health system model of health professional consultations, may lead to more successful weight control in pre-menopausal women than a self-directed intervention based on health education messages. The results of this trial will significantly add to the body of literature on methods of obesity prevention at menopause, which is an under-researched area.

The 40-Something study is also an innovative application of MI to prevent obesity, rather than treat it. By using a Dietitian and an Exercise Physiologist in applying the counselling framework to increase RCT effectiveness, the 40-Something study has implications for skills development of health professionals in practice settings. This RCT addresses some of the short-falls of the only other reported menopausal weight control intervention in that it tests a lower intensity intervention. Importantly, it will also ascertain whether the current healthcare system can support the delivery of effective menopausal weight prevention programs to address this significant public health issue.

Limitations of this review include the use of self-reported data for the weighed food record and the physical activity diaries relating to the issue of misreporting and social desirability response. It was not possible to achieve participant blinding for this study, increasing the risk of bias, though efforts were made to achieve researcher blinding. As with any measures, the tools we used are subject to bias and the TFEQ in particular requires further validation in a mid-age population. The compliance measurement at three months will likely be subject to social desirability bias given the diary recording, although the pedometers were included in an attempt to objectify the measurement of physical activity. The stored blood samples will allow for future antioxidant analysis as a validation of the four-day weighed food record data. The sample size meant the study was underpowered to assess differences in the secondary outcome variables, but it was sufficiently powered to detect weight differences according to the goals for each weight category. Study findings from phase 1 will be collated in 2012 and from phase 2 in 2013 and, if shown to be feasible, will help to inform the design of a larger randomized controlled trial to determine intervention effectiveness in preventing metabolic syndrome, and cost effectiveness of the intervention.

## Endnote

^a^ The amount of 5 kg was chosen given it is approximately 7% of 73 kg which was assumed to be the mean baseline weight for the overweight women.

## Competing interests

The authors declare that they have no competing interests.

## Authors’ contributions

LTW, JLH, CEC and PJM were responsible for the study design. All authors were responsible for drafting and revising the manuscript and have approved the final version. All authors read and approved the final manuscript.

## Pre-publication history

The pre-publication history for this paper can be accessed here:

http://www.biomedcentral.com/1471-2458/13/1007/prepub

## Supplementary Material

Additional file 1 Goals for Weight according to BMI. Click here for file

Additional file 2 Intervention components (Phase 1) of the 40-Something RCT. Click here for file

Additional file 3 Procedure for each data collection event. Click here for file
