# Supplementary figures and images for: The 40-Something randomized controlled trial to prevent weight gain in mid-age women
Source: BMC Public Health. 2013 Oct 25;13:1007. doi: 10.1186/1471-2458-13-1007 (PMC4016250; doi:10.1186/1471-2458-13-1007)

## Additional file 1

### Goals for Weight according to BMI

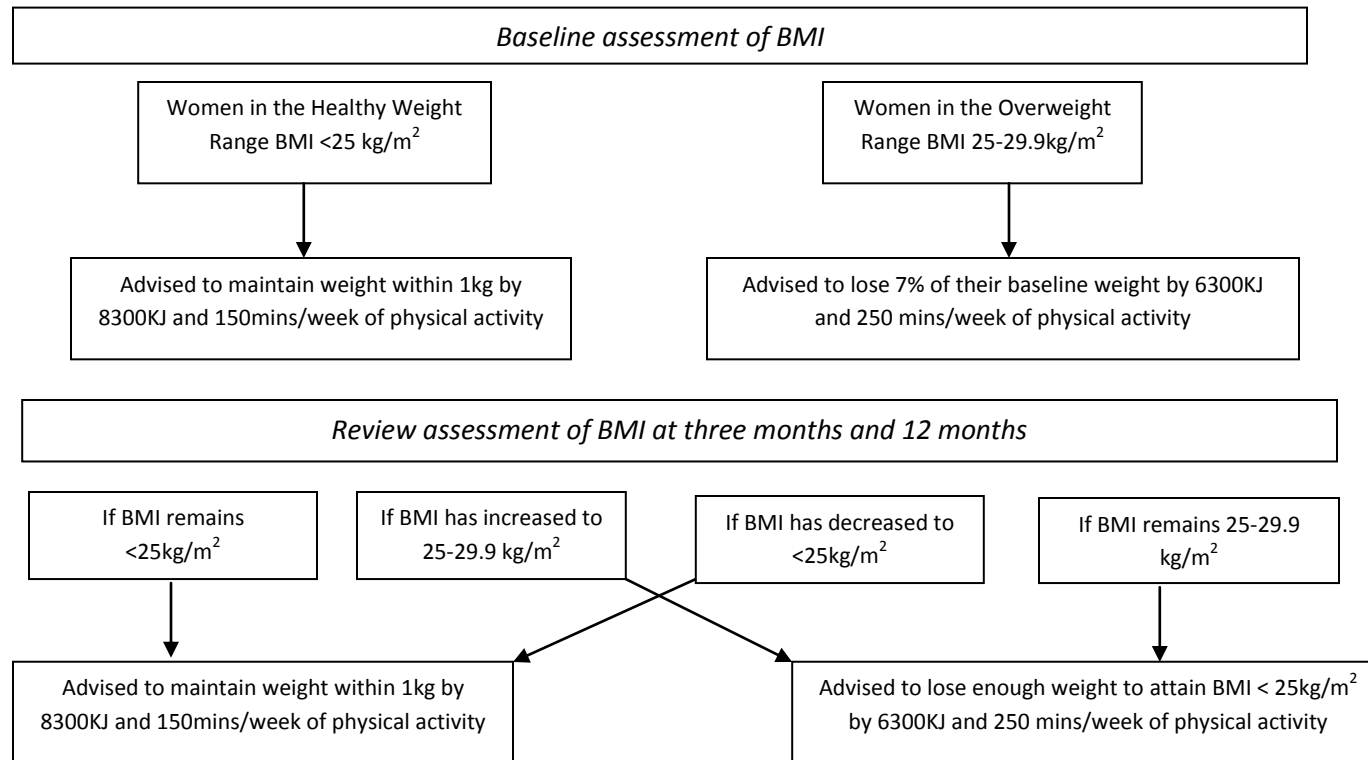

Supplement: Additional file 1 — Goals for Weight according to BMI. [file 1471-2458-13-1007-S1.pdf]
