# Supplementary material for: The 40-Something randomized controlled trial to prevent weight gain in mid-age women
Source: BMC Public Health. 2013 Oct 25;13:1007. doi: 10.1186/1471-2458-13-1007 (PMC4016250; doi:10.1186/1471-2458-13-1007)
Supplement: Additional file 2 — Intervention components (Phase 1) of the 40-Something RCT. [file 1471-2458-13-1007-S2.pdf]

Additional File 2: Intervention components (Phase 1) of the 40-Something RCT

Component	*Rationale for inclusion*	*MI*	*SDI*
Written feedback or measures of health status at baseline, 3 and 12 months (BP, anthropometry blood).	Basis of goal setting<br>Motivation for participants	Provided as part of consultation with Dietitian at consultations 1 (baseline measures), 3 (compliance measures) and at 12 month measures appointment (provided in person except for blood results which were mailed)	Provided by mailout with a covering letter.<br>- Mailout 1 after baseline measures<br>- Mailout 2 after 3 month measures<br>- Mailout 3 after 12 month measures
Written feedback on dietary intake and physical activity at baseline, 3 and 12 months.	Basis of recommendations for behaviour change<br>Motivation for participants.	As above.	As above.
Written weight goals and strategies to change diet and physical activity after baseline and 3 months measures.	Clear goals and strategies.	Negotiated with participant by Dietitian as part of consultations.	Provided as part of mailouts 1 and 2.
12 page weight control brochure for weight loss (if BMI 25-29.9) or prevent weight gain (if BMI 18.5-24.9). Diet and physical activity goals clearly stated. Weight loss: 6300kJ and 250 minutes physical activity per week.	Contained 10 weight control messages for participants to refer back to.<br>Detailed how to put dietary and physical activity change in to practice (e.g. daily meal plan, recipes, tips to increase incidental activity).	Handed to participants during consultation 1.	Sent by post as part of mailout 1.
Self-monitoring tool: Weight tracking recording sheet, physical activity and diet dairy, given to all participants.	Self-monitoring of weight and associated behaviours has been shown to improve compliance and weight change outcomes[50-52]	Materials given in consult 1. Progress with self-monitoring evaluated in consultations 3,4 and 5	Materials sent by post as part of mail out 1. Self-monitoring was encouraged in the Newsletter.
Menstrual record sheet.	Used to check menopause status	Handed to participants upon attending baseline and 12 month assessments	Handed to participants at baseline and 12 month assessments

40-Something Newsletter 4xA4 sheets	Prevent attrition by promoting study involvement to participants, provide recipe ideas and encourage self-monitoring	Mailed to participants at 6 monthly intervals.	Mailed to participants at 6 monthly intervals.
Consultation 1: with Accredited Practising Dietitian applying MI principles of discrepancy development to negotiate goals and 3 behaviour change strategies for dietary change based on analysis of 4 day record.	By establishing discrepancy between actual and desired behaviour in eating, specific goals and strategies are set by the participant.	Aimed at guiding the participant to eat a high nutritional quality diet that met their relevant daily energy intake goal (8300kJ or 6300kJ) and nutrient requirements [30]; and adhered to the seven diet messages.	×
Consultation 2: with Exercise Physiologist applying MI principles to negotiate 3 strategies for physical activity based on 4 day record and after screening for prior injury.	By establishing discrepancy between actual and desired behaviour in physical activity, specific goals and strategies can be set by the participant.	Participants were encouraged to evaluate their compliance with the study's weight control messages around activity and inactivity.	×
Consultation 3: with Dietitian – 3 months. Compliance with weight goal and 3 dietary and 3 physical activity strategies and control messages were checked. Barriers to change and ways to overcome these barriers were discussed. Special topic: emotional eating.	Discrepancy focus was in resolving body dissatisfaction.	Feedback provided and goals and strategies revised where necessary.	×
Consultation 4: with Dietitian – 6 months. As for session 3 with special topic: understanding nutrition information panels.		Feedback provided and goals and strategies revised where necessary.	×
Consultation 5: with Dietitian – 9 months. As for session 3 plus special topic: staying motivated. Discrepancy focus: resolving body dissatisfaction.	Need to maintain motivation during maintenance phase	Decisional balance activity.	×
