# Supplementary material for: The 40-Something randomized controlled trial to prevent weight gain in mid-age women
Source: BMC Public Health. 2013 Oct 25;13:1007. doi: 10.1186/1471-2458-13-1007 (PMC4016250; doi:10.1186/1471-2458-13-1007)
Supplement: Additional file 3 — Procedure for each data collection event. [file 1471-2458-13-1007-S3.pdf]

Additional file 3 : Procedure for each data collection event

Station	***Baseline***	***3 mo***	***12 mo***	***18 mo***	***24 mo***
1. Greeting participants admitted and procedure explained.	✓	✓	✓	✓	✓
Gave questionnaire to complete while moving around stations.	✓		✓		✓
2. Blood pressure measurement	✓	✓	✓		✓
3. Height	✓		✓		✓
4. Weight	✓	✓	✓	✓	✓
5. Body composition –					
a. BIA	✓	✓	✓	✓	✓
b. Inbody Analyser*			✓	✓	✓
6. Waist circumference	✓	✓	✓	✓	✓
7. Blood collection by trained phlebotomist	✓		✓		
8. Breakfast provided	✓	✓	✓	✓	✓
Questionnaires completed	✓		✓		✓
9. Instructed in how to complete 4 day diet and physical activity diary and given scales and pedometer after checking equipment	✓	✓	✓		
10. Questionnaires checked for completeness.	✓		✓		✓
Explained next stage and discharged	✓	✓	✓	✓	✓
